# A successful treatment-free remission is achievable also by chronic myeloid leukemia patients lacking optimal requirements

**DOI:** 10.1038/s41408-024-01025-7

**Published:** 2024-03-26

**Authors:** Massimiliano Bonifacio, Luigi Scaffidi, Maria Cristina Miggiano, Davide Facchinelli, Luca Tosoni, Sara Pezone, Davide Griguolo, Giulia Ciotti, Marco Danini, Andrea Bernardelli, Rita Bresciani, Monica Cavraro, Lara Crosera, Elena De March, Michele Dell’Eva, Laura Dorotea, Luca Frison, Lara Furlani, Ilaria Gianesello, Ester Lovato, Elena Marchetti, Gianluca Morelli, Rikard Mullai, Umberto Pizzano, Simone Zoletto, Renato Fanin, Mauro Krampera, Livio Trentin, Elisabetta Calistri, Giuseppe Carli, Gianni Binotto, Mario Tiribelli

**Affiliations:** 1 Department of Engineering for Innovation Medicine, Section of Innovation Biomedicine, Hematology Area, University of Verona and Azienda Ospedaliera Universitaria Integrata, Verona, Italy; 2 Department of Hematology, San Bortolo Hospital, Vicenza, Italy; 3 Division of Hematology and BMT, Azienda Sanitaria Universitaria Friuli Centrale and Department of Medical Area, University of Udine, Udine, Italy; 4 Department of Medicine, Hematology and Clinical Immunology, University of Padua, Padova, Italy; 5 Hematology Unit, Azienda Sanitaria Universitaria Giuliano-Isontina, Trieste, Italy; 6 Onco Hematology, Department of Oncology, Veneto Institute of Oncology, IOV-IRCCS, Padova, Italy; 7 Hematology Unit, Ca’ Foncello Hospital, Treviso, Italy; 8 Department of Medicine, Oncology Unit, San Donà di Piave (VE), Italy; 9 Hematology Unit, San Martino Hospital, Belluno, Italy; 10 Hematology and Bone Marrow Transplant Unit, Ospedale Regionale San Maurizio, Bolzano, Italy; 11 Oncology Unit, Ospedali Riuniti Padova Sud-Schiavonia, Monselice (PD), Italy; 12 Hematology Unit, Ospedale Dell’Angelo, Mestre-Venezia, Italy; 13 Oncology Unit, Ospedale Mater Salutis, Legnago (VR), Italy

**Keywords:** Chronic myeloid leukaemia, Risk factors, Myeloproliferative disease

The goal of achieving sustained treatment-free remission (TFR) after discontinuation of tyrosine kinase inhibitors (TKI) is of increasing importance in the management of chronic myeloid leukemia (CML) [1]. Different variables at diagnosis and, more importantly, upon response to treatment have been associated with distinct probabilities of a successful TFR. In an effort to expand access to TFR from the clinical trial setting to the wider clinical practice, “optimal” requirements which definitely prompt for treatment discontinuation have been proposed [2, 3]. However, there is a lack of information about the outcome of patients who discontinue TKI treatment when one or more of these optimal requirements are missing.

Within a retro-prospective registry started in 2014 in the Triveneto region (North-East of Italy), we studied a series of CML patients who discontinued TKIs from 2011 to 2023 outside of clinical trials. Only patients with less than MR^4.0^ at TKI stop were excluded from the analysis. We defined as potential risk factors for discontinuation the followings: (a) high ELTS risk, (b) history of accelerated/blast phase (AP/BP), (c) warning or failure molecular responses (according to ELN2020 definitions) at 3, 6 and/or 12 months of the TKI later discontinued, (d) switch to second-line TKI for resistance, (e) total duration of treatment ≤5 years, (f) “non-optimal” duration of deep molecular response (DMR) before TKI stop, defined as ≤2 years if MR^4.5^ or as ≤3 years if MR^4.0^, (g) use or ≥3 lines of TKI, and (h) history of BCR::ABL1 mutations. The endpoint of the study was the rate of sustained TFR (i.e., at least MR^3.0^ without further treatments) 12 months after TKI discontinuation depending on the number of risk factors.

According to the inclusion criteria, 236 patients were retrieved in our registry. Males were 124 (52.5%), median age at CML diagnosis was 50.4 years (range 17–80), and median age at TKI stop was 61.7 years (range 23–91). All patients had typical e13a2 and/or e14a2 BCR::ABL1 transcript type. All patients were diagnosed in chronic phase (CP), except one in AP (due to 12% blasts at diagnosis). ELTS risk was low/intermediate/high/unknown in 162 (68%)/54 (23%)/9 (4%)/11 (5%) patients, respectively.

Frontline treatment was imatinib in 201 (85%) and 2G-TKI in 35 (15%) patients. Most patients (*n* = 172, 73%) received only one line of TKI treatment, while 54 (23%) and 10 (4%) received 2 or ≥3 lines of treatment, respectively. Reasons for first-line TKI stop were elective TFR in 162 (69%), toxicity in 32 (13%), resistance in 35 (15%), and other (e.g., pregnancy) in 7 (3%) patients.

At discontinuation, 144 patients (61%) were receiving imatinib, 58 patients (25%) nilotinib, 30 patients (13%) dasatinib, while bosutinib and ponatinib were taken by 3 and 1 patients, respectively. Furthermore, at the time of discontinuation 114 patients (48%) were receiving TKI at full dose and 122 (52%) at a reduced dose: the probability of taking full or reduced dose did not differ among TKIs. Reasons for TKI discontinuation were elective TFR in 214 (91%), adverse events in 15 (6%), and other in 7 (3%) patients. Patients who discontinued TKI due to adverse events were taking imatinib (*n* = 7, all in first line), dasatinib (*n* = 5, of whom 1 in first line, 3 in second-line, and 1 in third-line) or nilotinib (*n* = 3, all in second-line).

We categorized the kinetics of response considering the last TKI used by each patient, i.e., the one subsequently discontinued: out of 196 with available information at 3, 6, and 12 months after TKI start, 156 (80%) achieved an optimal response to all timepoints while 40 (20%) patients had at least one non-optimal response, mainly warning at 6 and/or 12 months.

Four patients had a history of BCR::ABL1 mutations, all emerged during frontline imatinib treatment: two patients, harboring E352G and A399M mutations respectively, were switched to nilotinib, and two patients, harboring E255K and F359C mutations respectively, were switched to dasatinib. All these patients rapidly cleared the mutations and discontinued their second-line TKI after a median TKI duration of 6.4 years (range 4.1–10.2) and a median DMR duration of 3.4 years (range 1.8–6.5).

The median total duration of TKI treatment was 9.1 years (range 1.3–19.4); specifically, 216 patients (91%) were treated for >5 years while 20 patients (9%) received ≤5 years of TKI treatment: in these 20 patients median duration of treatment was 4.1 years (range 1.3–5.0) and reasons for discontinuation were elective TFR in 17 (85%) and adverse events in 3 (15%) patients. The median duration of DMR before TKI stop was 5.15 years (range 0.1–15.7) for the entire cohort and was 4.9 years (range 0.6–14.6) and 5.3 years (range 0.1–15.7) for patients with stable MR^4.0^ and stable MR^4.5^, respectively. Duration of DMR was “optimal” in 193 (82%) and “non-optimal” in 39 (17%) patients (for 4 patients the duration of stable DMR was not estimable due to lack of regular measurements): of these 39 patients, 31 (79%) had a stable MR^4.0^ with a median duration of 2.2 years (range 0.6–2.9), while 8 (21%) had a stable MR^4.5^ with a median duration of 1 year (range 0.1–1.9). Moreover, 8 patients had a median duration of DMR less than 1 year and reasons for discontinuation were toxicity in 6 cases and patient’s request for desired pregnancy in 2 cases.

Overall, patients without risk factors for discontinuation were 123 (52%), while patients with 1, 2, 3, 4 or 5 risk factors were 71 (30%), 29 (12%), 11 (5%), 1 (0.5%) and 1 (0.5%), respectively. Due to the low numbers, patients with 3, 4 or 5 risk factors were categorized together.

Median follow-up after discontinuation was 20.9 months (range 1–118) for the whole cohort and 39.1 months (range 1–118) for patients not losing molecular response in TFR. Patients with ongoing TFR but less than 12 months of follow-up after discontinuation were 32. The probability of sustained TFR at 12 months for the whole cohort was 73.8% (IC95% 67.5–79.1), not different between patients taking TKI at full or reduced dose (74.7% vs 72.9%, respectively). The rate of sustained TFR was 80.9%, 67.3%, 70.5% and 50.3% for patients with 0, 1, 2, and ≥3 non-optimal requirements, respectively (Fig. 1). Interestingly, patients who displayed resistance to frontline treatment (imatinib in all cases) and then attained a stable response with other TKIs had the same probability of successful TFR as patients who received only one line of TKI treatment and electively discontinued it (67% vs 76%, respectively). However, all the 4 patients who failed frontline treatment due to the presence of BCR::ABL1 mutations lost MR^3.0^ after a median of 5.7 months (range 2.5–9.2) from TKI discontinuation, despite an optimal kinetics of response to second-line treatment in all cases. At TFR failure the presence of BCR::ABL1 mutations was investigated in 3 out of 4 patients and all of them were negative. The single patient diagnosed in AP received frontline dasatinib (140 mg daily), attained an optimal response at all timepoints, and tried to suspend TKI after 6 years of undetectable molecular response, but rapidly relapsed after TFR start. The only variable associated to a significantly different probability of TFR success was duration of DMR (Table 1): patients with optimal and non-optimal duration had a 12-month TFR rate of 78.2% and 55.8% respectively (*p* = 0.001). There were no differences between the TFR success rate of patients with non-optimal duration of MR^4.0^ and non-optimal duration of MR^4.5^ (51.4% vs 62.5%, respectively, *p* = 0.27). Among the 66 patients who lost MMR, all except one restarted treatment with the same (*n* = 55) or a different TKI (*n* = 10): all regained MR^3.0^ in a median time of 2.8 months. No progression were recorded.

**Fig. 1:**
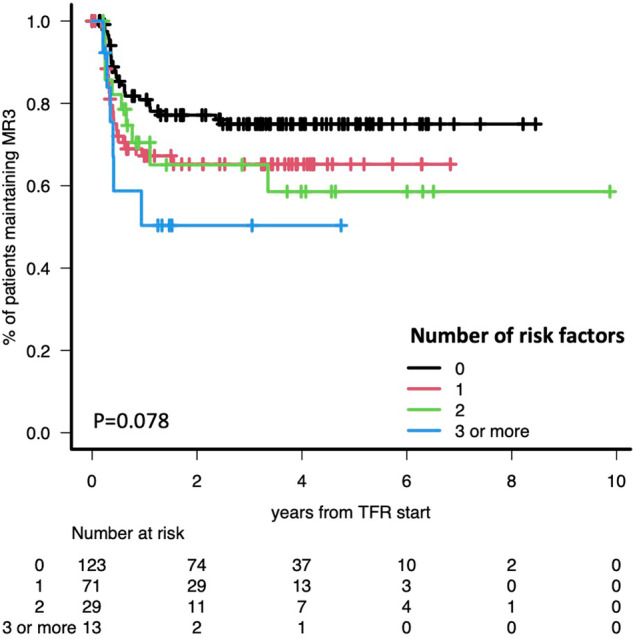
Rate of sustained TFR after TKI discontinuation according to the number of risk factors.

**Table 1:** Probability of maintaining TFR at 12 months after discontinuation according to the patients’ characteristics.

	*N*	12-month TFR rate	95% CI	*P*
ELTS risk				
Non-high	216	73.9%	67.3–79.4	0.451
High	9	62.5%	22.9–86.1	
Molecular response				
Optimal at all timepoints	156	70.9%	62.9–77.6	0.943
At least one non-optimal	40	74.7%	56.9–86.0	
Reason for frontline TKI stop				
Elective TFR	162	76.3%	68.7–82.3	0.472
Toxicity	32	62.8%	42.8–77.5	
Resistance	35	67.4%	48.6–80.5	
Other	7	100%	NA	
Total duration of TKI treatment				
>5 years	215	74.2%	67.6–79.7	0.455
≤5 years	20	73.3%	46.8–88.1	
Duration of DMR before TKI stop				
Optimal	193	78.2%	71.4–83.6	0.001
Non-optimal	39	55.8%	38.7–69.8	
Number of lines of TKI treatment				
1	172	75.8%	68.5–81.7	0.528
2	54	68.0%	53.0–79.1	
3 or more	10	68.6%	30.5–88.7	
Number of “TFR risk factors”				
0	123	80.9%	72.4–87.0	0.078
1	71	67.3%	54.6–77.1	
2	29	70.5%	49.4–84.1	
3 or more	13	50.3%	21.1–73.9	

The contemporary management of CML should be aimed at maximizing the rate of access to a successful TFR for virtually all patients [4]. Since the survival of patients treated with long-term TKIs is similar to that of the general population, high priority has been given to guarantee the safety of discontinuation, avoiding access to categories of patients for whom the risk of losing the response or even of undergoing progression was considered to be higher. The optimal requirements for TFR success proposed by the European LeukemiaNet recommendations [3] were based on the prognostic factors identified by earlier TFR trials [5]. However, the weight of individual variables has not been extensively studied. Recently, a large international retro-prospective study showed that the probability of progression to blast phase was near to 0% in a population of patients eligible to treatment discontinuation irrespective whether or not they actually suspended the therapy [6].

In the framework of a regional registry established in 2014 to assess the management of CML patients in the clinical routine, we analyzed the outcome of TFR according to the presence and the number of non-optimal requirements, which we defined as “risk factors” for TFR.

In addition to those we have selected, additional variables affecting the success of TFR have been proposed in the literature, including gender [7] and the transcript type [8]. However, given the lack of agreement on their prognostic impact and the possibility that the differences of molecular response between transcript types might partly depend on a technical bias [9], we decided not to include them in our model.

Overall, the rate of successful TFR at 12 months in our cohort (73.8%) was higher as compared to the EuroSKI study (56%) [10]. This could be due to the different length of total TKI treatment (9.1 years in our study, 7.5 years in the EuroSKI) but it is in line with studies demonstrating a superior TFR success in real-life studies than in clinical trials [6, 11, 12]. Therefore, even though the presence of non-optimal requirements progressively reduced the chance of maintaining molecular remission after discontinuation, the rate of TFR success was around 50% also in the most unfavorable group of patients, i.e., those lacking ≥3 optimal requirements, a figure that still justifies a TFR attempt.

In agreement with the findings from EuroSKI and many other studies [1, 10, 12] we found that the duration of stable DMR was the most relevant prognostic factor for TFR success. In a large real-life cohort of 284 patients, the duration of MR^4.0^ or MR^4.5^ ≥5 years was the only variable associated to a superior TFR success, with and estimated 5-year TFR rate of 87% and 92%, respectively [12]. Interestingly, also in our cohort duration of DMR ≥5 years vs <5 years were associated to significantly different chances of TFR success (81.9% vs 65.6%, respectively, *p* = 0.005).

We could not confirm the negative predictive value of high ELTS risk [13], however there were only 9 high-risk patients in our cohort, suggesting that many clinicians may be reluctant to offer TFR to this category of patients.

Data about TKI discontinuation after ≥2 lines of treatment are quite limited, especially when the switch is not due to intolerance, as some studies have shown that previous resistance to TKI is associated with a higher rate of relapse after discontinuation [6, 14]. Interestingly, in our study the probability of a successful TFR was identical in patients who discontinued electively frontline therapy and in those who received subsequent lines of therapy due to intolerance or resistance.

Claudiani et al. showed a 50% probability of successful TFR in a cohort of 10 patients with a history of BCR::ABL1 mutations [15], while all our 4 cases with a history of BCR::ABL1 mutations failed TFR.

The actual weight of BCR::ABL1 mutations and AP history on subsequent outcome of discontinuation should be confirmed in larger series.

We recognize that our observations may not be generalizable since the decision to allow TFR may have been influenced by clinicians’ feels and that not all patients lacking optimal requirements were offered to stop treatment. Due to this potential selection bias we believe that our data should not prompt clinicians to imprudently offer TFR option to all CML patients, however the prospective nature of the registry and the inclusion of all the patients who actually discontinued TKI while in DMR allow to examine how the decision of attempting TFR has been evolved over years in clinical practice.

We conclude that all CP CML patients may have access to a successful TFR even when non-optimal features are present, provided that there is an optimal duration of DMR before TKI stop and that close molecular monitoring is guaranteed, especially during the first months of TFR [3]. Our group recently showed that BCR::ABL1 value at 1 month after stop is strongly predictive of subsequent maintenance of TFR, and this value may be used to safely model the frequency of monitoring during TKI discontinuation [16].

## Data Availability

Aggregated data that underlie the results reported in this work will be available upon reasonable request. Patient-level data will not be shared.
